# Warburg effect, hexokinase-II, and radioresistance of laryngeal carcinoma

**DOI:** 10.18632/oncotarget.13044

**Published:** 2016-11-03

**Authors:** Jiang-Tao Zhong, Shui-Hong Zhou

**Affiliations:** ^1^ Department of Otolaryngology, The First Affiliated Hospital, College of Medicine, Zhejiang University, Hangzhou, Zhejiang, China

**Keywords:** hexokinase-II, Warburg effect, laryngeal carcinoma, radioresistance

## Abstract

Radiotherapy is now widely used as a part of multidisciplinary treatment approaches for advanced laryngeal carcinoma and preservation of laryngeal function. However, the mechanism of the radioresistance is still unclear. Some studies have revealed that the Warburg effect promotes the radioresistance of various malignant tumors, including laryngeal carcinoma. Among the regulators involved in the Warburg effect, hexokinase-II (HK-II) is a crucial glycolytic enzyme that catalyzes the first essential step of glucose metabolism. HK-II is reportedly highly expressed in some human solid carcinomas by many studies. But for laryngeal carcinoma, there is only one. Till now, no studies have directly targeted inhibited HK-II and enhanced the radiosensitivity of laryngeal carcinoma. Accumulating evidence has shown that dysregulated signaling pathways often result in HK-II overexpression. Here, we summarize recent advances in understanding the association among the Warburg effect, HK-II, and the radioresistance of laryngeal carcinoma. We speculate on the feasibility of enhancing radiosensitivity by targeted inhibiting HK-II signaling pathways in laryngeal carcinoma, which may provide a novel anticancer therapy.

## INTRODUCTION

Approximately 157,000 new cases of laryngeal carcinoma were diagnosed worldwide in 2012 (1% of all cancers), and approximately 83,400 people were estimated to have died from laryngeal carcinoma in that year (1% of all cancer deaths) [1]. Previously, total laryngectomy was the mainstay of treatment for laryngeal carcinoma, which was successfully performed for the first time in 1873 by Theodor Billroth [2]. Although this procedure decreases the recurrence rate of laryngeal carcinoma, it leaves patients with postoperative aphonia and reliance on a permanent tracheostoma. Nowadays, with the evolution of treatment for advanced laryngeal carcinoma, the focus of treatment has shifted to maintaining locoregional control while also maintaining a functional larynx [3]. Therefore, multidisciplinary treatment approaches are recommended. As radiotherapy (RT) techniques have markedly improved recently, the incidence of laryngeal function preservation in laryngeal carcinoma has increased [4–7]. However, RT results in acute toxicities including mucositis, dysphagia, xerostomia, dermatitis, and pain. In addition, radioresistance has been found in laryngeal carcinoma. However, the exact mechanisms behind this radioresistance have not been clarified. The mechanisms may include cancer stem cells [8–11], hypoxia [e.g., Hypoxia-inducible factor 1α; (HIF-1α)] [12], human papillomavirus (HPV) [13], and signal transduction pathways [e.g., the signal transducer and activator of transcription 3 (STAT3), phosphoinositide-3 kinase (PI3K)/Akt].

In recent years, the Warburg effect (aerobic glycolysis) has been widely investigated. It is an anomalous characteristic of cancer cells, in which they consume a large amount of glucose and favor aerobic glycolysis over oxidative phosphorylation even in the presence of oxygen. A few studies have demonstrated that the Warburg effect contributes to the radioresistance of various malignant tumors [12, 14–20]. Inhibition of the Warburg effect has been shown to enhance the radiosensitivity of cancers [14, 21–23]. Similar results have also been found in laryngeal carcinoma [24–29].

Hexokinase-II (HK-II) is the enzyme that catalyzes the first essential step of glucose metabolism, the conversion of glucose into glucose-6-phosphate, in the Warburg effect [30]. HK-II has been found highly expressed in many different cancers {e.g., laryngeal carcinoma [31], oral squamous cell carcinoma [32], hepatocellular carcinoma (HCC) [33, 34], breast cancer [35], colorectal tumors [36], etc.}. Several studies have demonstrated that HK-II upregulation not only increases the glycolysis rate, but is also required for tumor initiation and maintenance [37]. For example, voltage-dependent anion channel (VDAC)-bound HK-II contributes to the inhibition of apoptosis by suppressing the formation of mitochondrial permeability transition pores (mPTPs) [38]. On the other hand, numerous studies have indicated that inhibitors [e.g., 2-deoxyglucose (2-DG), lonidamine, 3-bromopyruvate (3-BP)] targeted inhibiting HK-II can induce cancer cells to undergo apoptosis [39]. To date, only one study has reported HK-II overexpression in laryngeal carcinoma [31], while another revealed that HK-II depletion sensitizes radioresistant Hep-2 cells to radiation, although the result did not reach significance [40]. As such, the association between HK-II and radioresistance in laryngeal carcinoma requires further investigation.

In this review, we discuss the role of the Warburg effect and HK-II in the radioresistance of laryngeal carcinoma, and the feasibility of enhancing radiosensitivity by targeted inhibiting the signaling pathways of HK-II in laryngeal carcinoma, which may be a novel anticancer therapy.

## RADIORESISTANCE OF LARYNGEAL CARCINOMA

Many studies have demonstrated that cancer stem cells, signal transduction pathways, and hypoxia are vital for the radioresistance of laryngeal carcinoma [26, 41–50]. These roles have also been reported in our previous reviews [25, 51]. Additional factors involved in radioresistance have also been identified, including changes in DNA levels and ultrastructure.

With regard to changes in DNA levels potentially mediating the radioresistance of cancer cells, Kim et al. found that the promoter-CpG islands of five previously identified radioresistance-related genes (TOPO2A, PLXDC2, ETNK2, GFI1, and IL12B) were significantly altered in radioresistant laryngeal carcinoma. This was determined by examining the differences in DNA methylation between control and radioresistant laryngeal carcinoma, the latter of which had been established by long-term fractionated irradiation. In addition, the demethylation of these gene promoters *via* a DNA methyltransferase inhibitor (5-aza-2′-deoxycytidine) was shown to increase their transcription levels. This study suggests that radiation-induced epigenetic changes can stimulate the radioresistance of laryngeal carcinoma [52]. Furthermore, the PAG1 gene has also been identified as a promising novel radiosensitization target for laryngeal carcinoma. Ke et al. used small interfering RNA (siRNA) to targeted suppress the PAG1 gene in a radioresistant cell line and dramatically enhanced its radiosensitivity and irradiation-induced cell death, while the ectopic expression of PAG1 in radiosensitive cell lines led to radioresistance and suppressed irradiation-induced cell death. These results demonstrate that PAG1 acts as a radioresistance factor in laryngeal carcinoma cells [53].

In a recent study, Yang et al. identified ultrastructural changes in the radiation-induced radioresistant laryngeal carcinoma Hep-2 (Hep-2R) cell line. Specifically, they observed increased nuclear atypia, more rough endoplasmic reticulum (ER), and fewer mitochondria in Hep-2R cells, which showed significant resistance to radiation compared with parental Hep-2 cells. This study indicated that ultrastructural changes are the morphological mechanism by which the radioresistance of Hep-2R cells is enhanced [54].

## AGENTS TARGETED INHIBITING THE HK-II-MEDIATED WARBURG EFFECT

Some studies have demonstrated that agents targeted inhibiting HK-II may have an anticancer effect.

### 2-DG

2-DG is a glucose analogue and a competitive inhibitor of HK-II. It is phosphorylated by HK-II but not further processed by glucose 6-phosphate isomerase, resulting in the accumulation of phosphorylated 2-deoxyglucose (P-2-DG) in the cell and its competitive inhibition of HK-II by negative feedback. 2-DG has reached the phase I/II clinical trial stage for the treatment of human cerebral gliomas [55] and phase I clinical trials for prostate cancer patients [56]. Previously, Wang et al. found that the combination of 2-DG with molecule US597 (UA-4, a structurally modified version of ursolic acid) synergistically inhibited hepatoma cell proliferation by dual targeting of apoptosis and glycolysis [57]. Subsequently, they found that 2-DG may inhibit hepatocarcinogenesis in DEN-treated rats by restricting cancer cell metabolism [58].

### 3-BP

In addition to 2-DG, 3-BP, a halogenated analog of pyruvic acid, can also inhibit HK-II function in aerobic glycolysis, which promotes tumor cell death by inducing ER stress in human HCC cell lines. An early study showed that 3-BP exhibits a strong anti-glycolytic effect on rat mammary tumor cells implanted in rats [59].

For HCC, the inhibition of carbonic anhydrase-IX (CA-IX) enhances the effect of 3-BP by aggravating ER stress and activating Jun NH2-terminal kinase (JNK) [60]. Another study revealed that combination therapy with 3-BP and a protein disulfide isomerase inhibitor (bacitracin) may be therapeutically useful in HCC [61]. A more recent study demonstrated that 3-BP exerts anti-hepatoma effects, as shown by *in vitro* and *in vivo* analyses [62]. In addition, in multiple myeloma (MM) cells, in which HK-II was over-expressed, 3-BP promptly and substantially suppressed adenosine triphosphate production and induced cell death [63]. For endometrial cancer, 3-BP induced tumor necrosis *in vitro* and inhibited tumor growth *in vivo* [64]. It has also been reported that 3-BP induced apoptosis in MDA-MB-231 breast cancer cells by downregulating Mcl-1 through the PI3K/Akt signaling pathway, which is upstream of HK-II [65]. Recently, Gandham et al. also showed that liposomal 3-BP improved permeability, HK-II inhibition, and cytotoxicity in a multicellular spheroid model of human ovarian adenocarcinoma (SKOV-3) cells [66, 67]. However, because of its off-target toxicity, 3-BP has not yet been widely used for anticancer therapy.

### Lonidamine

Lonidamine is a new drug that interferes with mitochondrial functions, which can inhibit HK-II function. It may be a useful tool for adversely affecting tumors *via* aerobic glycolysis [68]. Lonidamine has been evaluated in a phase II study, in combination with diazepam, for recurrent glioblastoma multiforme (GBM) [69]. It has also been used in other clinical trials for breast cancer, ovarian cancer, lung cancer, and prostate adenoma.

**Figure 1:**
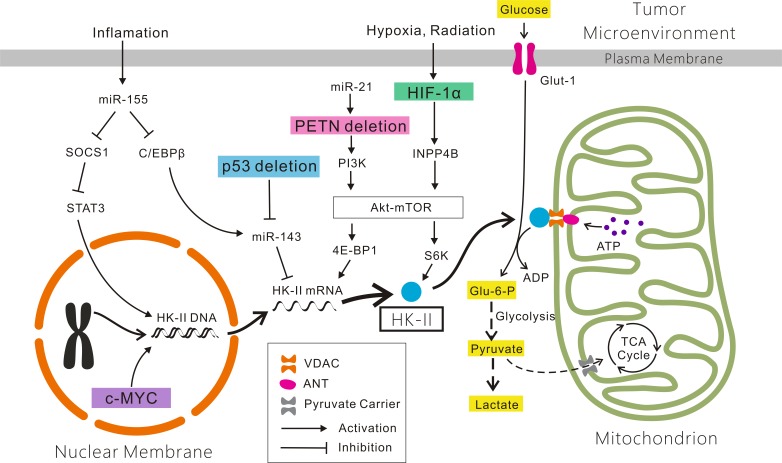
Mechanisms account for the altered expression of HK-II in cancer cells Glucose which across the plasma membrane *via* Glut-1 is phosphorylated by HK-II bound to VDAC located on the outer mitochondrial membrane. The ATP involved in the phosphorylation of glucose is transfered by ANT through the HK-II/VDAC complex. The product Glu-6-P enters into the pentose phosphate shunt for biosynthesis of nucleic acid precursors (not shown), and the glycolytic pathway. Most pyruvate is reduced to lactate and some is directed to mitochondria *via* the pyruvate carrier for the TCA cycle for energy generation and biosynthesis. The HIF-1α/INPP4B/Akt/mTOR/S6K, PTEN/PI3K/Akt/mTOR/4E-BP1, miR-155/SOCS1/STAT3, miR-155/(C/EBPβ)/miR-143, p53/miR-143 and c-MYC signaling pathways are showed here. See details in the text. Abbreviations: HK-II: Hexokinase-II; Glut-1: glucose transporter-1; VDAC: voltage-dependent anion channel; ANT: adenine nucleotide translocator; Glu-6-P: Glucose-6-P; TCA cycle: tri-carboxylic acid cycle.

### Targeted inhibiting the HK-II-VDAC complex

Mitochondrion-associated HK-II correlates strongly with the Warburg effect. The association between HK-II and the mitochondrial membrane is mediated by VDAC. The HK-II-VDAC complex functions not only in glycolysis and biosynthesis, but also in the inhibition of mitochondrion-induced apoptosis and the suppression of cell death. Conversely, inhibition of the HK-II-VDAC complex significantly enhances the induction of apoptosis in tumors [70]. The binding of HK to VDAC prevents the localization of other activated pro-apoptotic molecules (e.g., Bad, Bax) to the outer mitochondrial membrane and also prevents formation of the mPTPs complex, thereby avoiding mitochondrial cytochrome c-mediated apoptosis [71].

Recent studies have identified several agents that targeted inhibit the HK-II-VDAC complex, which is regarded as a promising mitochondrial target as it is linked to glycolysis, oxidative phosphorylation, and mitochondrion-mediated apoptosis in tumor cells [72]. 3-BP, which has been discussed above, dissociates HK-II from the HK-II-VDAC complex, causing tumor cell death. Some natural compounds have been shown to promote the detachment of HK-II from mitochondria, such as the natural plant flavonoid (oroxylin A), the plant hormones [methyl jasmonate (MJ), neoalbaconol (NA), and prosapogenin A] [73], and other synthetic compounds (casiopeina II-gly, clotrimazole, and bifonazole). Clotrimazole and bifonazole, which are azole derivatives, have been reported to sensitize glioblastoma cells to radiation by displacing HK-II from the mitochondrial membrane [74]. HK-II-targeted small hairpin RNA (shRNA) has also been shown to produce pronounced anti-tumor effects. The evidence above indicates that abrogation of the HK-II-VDAC complex may result in tumor cell apoptosis, thus increasing the sensitization of tumor cells to RT [38].

### Metformin

Metformin, an anti-hyperglycemic drug, has also been revealed to exhibit important anticancer properties. One study showed that it impairs the enzymatic function of HK-II in Calu-1 cells, thus abolishing cell glucose uptake and phosphorylation, which results in mitochondrial depolarization and subsequent cell death [75]. A similar phenomenon was also found in breast cancer [35].

### HK-II siRNA

siRNA have been used conventionally in basic research to silence target genes and observe the associated functional changes. Knockdown of HK-II with siRNA reduces its mRNA and protein levels, as well as its activity, resulting in cell cycle arrest at the G1 phase, attenuated glycolysis, and apoptosis in cancer cells [31, 76–78]. Silencing of the HK-II gene sensitizes human colon cancer cells to 5-fluorouracil [79] and sensitizes parental lung cancer cells to cisplatin [80]. In addition, the combination of HK-II interference and ^131^I therapy demonstrated a stronger anticancer effect in anaplastic thyroid carcinoma cells [81]. However, further investigations of treatments with HK-II siRNA for laryngeal carcinoma are required.

## THE WARBURG EFFECT AND RADIORESISTANCE

### The Warburg effect and radioresistance of cancer

The Warburg effect is significant for cancer progression. It provides a survival advantage and facilitates the synthesis of biosynthetic precursors required for continued cellular proliferation.

Bhatt et al. found that mitochondrial respiratory modifiers [e.g., dinitrophenol (DNP)] induced the transient stimulation of glycolysis in the malignant cell lines BMG-1 and OCT-1, conferring radioresistance to the cells. They also demonstrated that the radioresistance of cells induced by DNP was obtained by reducing residual DNA damage and cytogenetic damage linked to mitotic death *via* the non-homologous end joining and homologous recombination pathways [16]. Tumor hypoxia is a common phenomenon revealed to be an important effector promoting tumor cell survival. A clinical study demonstrated that hypoxia is associated with the radioresistance of prostate cancer [12]. A similar study showed that hypoxia and anaerobic metabolism may be important components of prostate cancer radioresistance [18]. Hypoxia-inducible factor 1 (HIF-1) is important in the Warburg effect; it becomes active not only under hypoxic, but also normoxic, conditions. Accumulating evidence shows that HIF-1 functions in the induction of radioresistant characteristics in cancer cells and tumor recurrence after radiation therapy [15]. In addition, glucose transporter-1 (Glut-1), which also correlates strongly with the Warburg effect, was found to be associated with the radioresistance of breast cancer cells [14]. Akt regulates multiple steps in the Warburg effect, including inducing Glut-1 gene expression and enhancing HK activity. Shimura et al. showed that an Akt-mediated process enhanced aerobic glycolysis in the acquisition of radioresistance by tumor cells [17]. Signal transducer and activator of transcription 1 (STAT1), which is usually regarded as a transmitter of interferon signaling and a pro-apoptotic tumor suppressor, has also been found to be associated with the Warburg effect. Pitroda et al. found that activation of STAT1 enhances the radioresistance of tumors [20]. Furthermore, radioresistance was found to be positively correlated with the lactate concentration [19]. It has been reported that rapamycin can decrease production of lactate, which reverses pseudo-hypoxic state and lactate acidosis [82, 83].

Targeted inhibiting the Warburg effect enhances the radiosensitivity of tumors. Bol et al. found that Warburg-phenotype tumor cells with impaired mitochondrial respiration were radiosensitive compared with wild-type parental cells [22]. Pyruvate kinase M2 isoform (PKM2) is a key regulator of the Warburg effect. It is expressed exclusively in cancers. Meng et al. showed that silencing PKM2 expression by shRNA enhanced radiation-induced apoptosis and autophagy in non-small cell lung cancer cell lines and xenografts [21]. Pitroda et al. identified new functions of STAT1, that its knockdown significantly induced radiosensitization of irradiated tumors, which indicated that STAT1 activation enhances the radioresistance of tumors [21]. Furthermore, mutation of tumor protein 53 (TP53) in head and neck squamous cell carcinoma (HNSCC) cells was found to be correlated with the Warburg effect. TP53 mutations were also correlated with HNSCC radioresistance. Sandulache et al. reported that the TP53 mutational status may be used as a marker to overcome radioresistance [29]. In addition, Pena-Rico et al. found that knockdown of TP53 induced the expression of a glycolysis and apoptosis regulator (TIGAR), resulting in radiosensitization of glioma cells [23]. The inhibition of Glut-1 has also been shown to sensitize radioresistant breast cancer cells to irradiation [14].

### The Warburg effect and radioresistance of laryngeal carcinoma

In a review, we reported that Glut-1 and the phosphatidylinositol 3-kinase/protein kinase B pathway are associated with cancer radioresistance [25]. In the case of laryngeal carcinoma, similar results have also been revealed. Finally, in a previous study, we found that hypoxia enhanced the radioresistance of CD133-positive Hep-2 cells [26]. Our previous studies also revealed that inhibition of Glut-1 expression by antisense oligodeoxynucleotides and the PI3K/Akt signaling pathway can improve the radiosensitivity of laryngeal carcinoma [27, 28]. Thus, we think that the Warburg effect may be linked to the radiosensitivity of laryngeal carcinoma.

## HK-II AND RADIORESISTANCE

### HK-II and radioresistance of cancer

HK-II is highly expressed in a variety of cancers. Tian et al. showed that HK-II was overexpressed within oral squamous cell carcinoma by HK-II immunohistochemical staining of tumor sections from 19 patients [32]. Increased HK-II expression was also seen in GBM [84]. Clinically, HK-II expression correlates with worse overall survival of GBM patients [85]. Similar results have also been shown in HCC, in which HK-II overexpression was induced by hypoxia [34, 86]. It was also shown that hypoxia-mediated HK-II induction enhanced mitochondrial stability. The inhibition of HK-II caused the release of this enzyme from the permeability transition pore complex, thus leading to the activation of mitochondrial apoptotic signals [33]. Another study showed that the combination of positive HK-II and negative phosphorylated pyruvate dehydrogenase-E1alpha (p-PDH) was associated with reduced recurrence-free survival of colorectal tumor patients [36]. In addition, for breast cancer, direct inhibition of the enzymatic function of HK-II using metformin caused evident tumor necrosis [35]. These results indicate that HK-II promotes tumor progression.

It has been revealed that HK-II is overexpressed after RT in prostate cancer patients [87]. And the depletion of HK-II increases the sensitivity of GBM cells to radiation [85], which suggested that the high expression of HK-II enhances the radioresistance of tumors.

### HK-II and radioresistance of laryngeal carcinoma

The high expression of HK-II has already been investigated in laryngeal carcinoma. Chen et al. assessed HK-II expression in laryngeal squamous cell carcinoma tissues by immunohistochemistry and found that it was significantly higher in this condition than in papilloma or glottis polypus. Stronger HK-II staining was also found to be associated with higher T, N, and TNM stages. This research group then inhibited HK-II expression in Hep-2 cells using shRNA and found that cells expressing shRNA demonstrated a higher G0:G1 ratio, increased apoptosis, and reduced viability. In addition, xenograft tumors derived from cells expressing shRNA were smaller and less proliferative than those from untransfected or control-plasmid-transfected cells [31]. This study suggests that HK-II promotes the development of laryngeal carcinoma.

Min et al. demonstrated that codepletion of Inositol polyphosphate 4-phosphatase type II (INPP4B) and HK-II notably sensitized radioresistant-Hep-2 cells to irradiation. In addition, HK-II depletion sensitized radioresistant-Hep-2 cells to radiation, although the result did not reach significance [40].

### Possible mechanisms of enhancing the radiosensitivity of laryngeal carcinoma *via* HK-II regulation

Accumulating evidence has shown that dysregulated signaling pathways often result in HK-II overexpression. Various molecular mechanisms, including genetic and epigenetic mechanisms, oncogenes, tumor suppressor genes, and microRNAs (miRNAs), have been suggested to account for the altered expression of HK-II in cancers.

#### HIF-1α is involved in the HK-II-mediated Warburg effect

HIF-1α is a subunit of a heterodimeric transcription factor, HIF-1, which is encoded by the HIF1A gene. HIF-1αis elevated in many different cancers. It is a major transcriptional regulator of the cellular metabolism response to hypoxia, which is common in the tumor microenvironment. The expression of HIF-1α was found to be positively correlated with the expression of HK-II in gastric adenocarcinoma [88]. The activation of HIF-1α under hypoxic conditions increases glycolysis by inducing glycolysis-regulatory genes including HK-II [89, 90]. A review has also indicated that HIF-1 enhanced the radioresistance of cancer cells through the reprogramming of glucose metabolism by increasing the expression of glycolytic enzymes, including HK-II [15].

INPP4B is a regulatory enzyme that selectively removes the phosphate from the fourth position of the inositol ring in phosphatidylinositol(3,4)-bisphosphate, which is involved in the phosphatidylinositol signaling pathway [91]. Kim et al. demonstrated that INPP4B is a novel marker of radioresistance of laryngeal carcinoma, which is highly expressed in radioresistant laryngeal carcinoma [47]. In addition to irradiation, hypoxia also induces INPP4B expression. Min et al. found that the level of INPP4B was induced under hypoxic conditions, concomitant with an increase in HIF-1α protein levels in laryngeal carcinoma Hep-2 cells. After transfection of siRNA against HIF-1α, both hypoxia- and radiation-induced INPP4B expression decreased, indicating that such expression is increased by HIF-1α in laryngeal carcinoma Hep-2 cells [41]. In addition, the association of INPP4B-mediated radioresistance with Akt survival signaling has been revealed [47]. In another study, silencing of INPP4B blocked the activation of Akt, while the overexpression of INPP4B increased proliferation and triggered the anchorage-independent growth of normal colon epithelial cells [92]. Stress-induced INPP4B expression exerted a cytoprotective effect by suppressing apoptotic cell death *via* upregulation of Akt activation.

The serine/threonine kinase Akt, also known as protein kinase B, has emerged as one of the most frequently activated protein kinases in human cancer. It has been referred to as a “Warburg kinase” [93]. Shimura et al. found that inhibition of the Akt pathway by API-2, an Akt inhibitor, eliminated the aerobic glycolysis enhanced by increased Glut-1 expression in HepG2 (human liver cancer) and HeLa (cervical cancer) cell lines, which exhibits radioresistance acquired by long-term exposure to fractionated radiation. They also found that inhibiting glycolysis using 2-DG, an HK-II inhibitor, suppressed acquired tumor cell radioresistance [17]. In fact, the activity of HK-II is thought to be induced by Akt. Akt activity promotes both the binding of HK-II to mitochondria and glucose uptake in cancer cells [94]. The mechanisms by which Akt induces the activity of HK-II have not been fully elucidated. However, in a recent review, Roberts and Miyamoto described that HK-II expression was upregulated by the Akt/mechanistic target of rapamycin complex 1 (mTORC1) pathway [95].

The Akt-mechanistic target of rapamycin (mTOR) pathway is strongly associated with increased glycolysis in tumors [96]. mTOR is considered an upstream activator of HIF-1α, EIF4E-binding protein 1 (4E-BP1), and S6 kinase (S6K), which are upstream activators of HK-II in the Warburg effect in cancer cells [40, 97]. mTOR is also a suppressor of some miRNAs, such as miR-143 and miR-125b, which are upstream suppressors of HK-II in the Warburg effect [98, 99]. In HNSCC-derived cell lines, miR-143 expression was inversely correlated with HK-II level. Furthermore, miR-143 was shown to inhibit HK-II expression *via* a conserved miR-143 recognition motif located in the 3′-untranslated region of HK-II mRNA [100]. This mechanism has also been demonstrated in lung cancer, colon cancer, and renal cell carcinoma [77, 98, 101]. In addition to miR-143, miR-125b targeted inhibits HK-II to sensitize human HCC cells to chemotherapy [99].

#### The role of suppressor oncogenes in the HK-II-mediated Warburg effect

Apart from HIF-1α, other signaling pathways have been proposed to be involved in the HK-II-mediated Warburg effect. Deficiencies in the tumor suppressor genes phosphatase and tensin homologue (PTEN) and p53 were found in the HK-II-mediated Warburg effect.

miRNA-21 (miR-21)/PTEN/PI3K/Akt/mTOR signaling pathway

The PTEN/Akt/mTOR pathway was the focus of a review by Steelman et al. It was shown to promote the growth and suppress the sensitivity of cancer to therapy [97]. PTEN is frequently inactivated in many human cancers *via* point mutations, among others. The deficiency or inactivation of PTEN leads to elevated Akt activity and unrestricted proliferation. The involvement of the PTEN/Akt/mTOR/4E-BP1 pathway in the HK-II-mediated Warburg effect was subsequently confirmed by Wang et al. in prostate cancer. HK-II protein expression is increased by PTEN deletion. This research group subsequently depleted or knocked down Akt or Raptor (an essential component of the mTORC1 complex) in PTEN/p53 double-deficient MEFs or the prostate cancer cell line UMN-4240P, resulting in a significant reduction in HK-II protein expression. In a later study, the pharmacological inhibition of Akt/mTORC1 signaling by NVP-BEZ235 was found to reduce HK-II protein expression remarkably. As BEZ235 significantly inhibited the activation of 4E-BP1, which is the primary downstream effector in the Akt-mTORC1 pathway, it was suggested that 4E-BP1 might increase HK-II expression [102].

Recently, it was demonstrated that inhibition of miR-21 in bladder cancer cell lines reduced glucose uptake, lactate production, and HK activity, which are considered to be related to the Warburg effect [103, 104]. Meanwhile, miR-21 inhibition promoted PTEN expression, decreased phosphorylated Akt, and deactivated mTOR [105]. Other studies identified PTEN as a target gene of miR-21 in colorectal cancer, non-small cell lung cancer, intrahepatic cholangiocarcinoma, ovarian cancer, cervical cancer, esophageal cancer, and HER2-positive gastric cancer [106–114]. It was also revealed that miR-21 expression was upregulated in laryngeal carcinoma, while PTEN expression was downregulated. The level of miR-21 was also found to be inversely correlated with PTEN expression [115].

PTEN has actually been reported to be an antagonist of PI3K signaling, which mediates the Akt/mTOR pathway. Hopkins et al. showed that a 576-amino-acid translational variant of PTEN (named PTEN-Long), which is a membrane-permeable lipid phosphatase secreted from cells that can enter other cells, antagonized PI3K signaling and induced tumor cell death *in vitro* and *in vivo* [116]. PI3K is a heterodimeric protein with an 85-kDa regulatory subunit and a 110-kDa catalytic subunit (PIK3CA). In an earlier study, the regulation of Akt by lipid products of PI3K was investigated by Franke et al. They found that Akt activity was correlated with the amount of phosphatidylinositol-3,4-bisphosphate (PtdIns-3,4-P2) *in vivo*, and synthetic PtdIns-3,4-P2 activated Akt both *in vitro* and *in vivo*. Akt is apparently activated by the direct correlation of PtdIns-3,4-P2 with the Akt PH domain [117]. Subsequently, Ahn et al. established SNU449 cells transfected with the HK-II gene using an expression vector. They found that the activated form of Akt was increased after transfection, and the PI3K inhibitor led to dissociation of mitochondrial HK-II into the cytoplasm. This revealed that HK-II promotes (18)F-FDG uptake and tumor proliferation *via* PI3K-dependent Akt signaling pathways [118]. The PI3K/Akt/mTOR signaling pathway was also reported to be involved in leukemogenesis [119]. Recently, it was reported that elevated HK-II expression was induced by activated PI3K/Akt signaling in osteosarcoma. Liu et al. found that the inhibition of PI3K/Akt signaling suppressed aerobic glycolysis, which could be reversed by reintroduction of HK-II [120].

p53/miR-143 pathway

Similarly to PTEN, another tumor suppressor gene, p53, was also found to be involved in the HK-II-mediated Warburg effect [90]. HK-II was selectively upregulated by the combined loss of PTEN and p53 in prostate cancer cells. In fact, p53 loss enhances HK-II mRNA stability through the inhibition of miR143 biogenesis [102], which, as discussed above, inhibits HK-II expression in various cancer cells [98]. It has also been revealed that the suppression of p53 activation by aldehyde reductase (AKR1A1) results in the acquisition of radioresistance by laryngeal carcinoma [46].

c-MYC-mediated high expression of HK-II

The oncogene c-MYC was positively correlated with the expression of HK-II. In fact, c-MYC regulates the transcription of genes involved in nearly every step of the glycolytic pathway, including HK-II, and enhances aerobic glycolysis [90]. Alvarez et al. demonstrated that c-MYC-driven tumors were characterized by high expression of HK-II and exhibited both high FDG uptake and rapid proliferation [121].

#### miR-155 upregulates HK-II through two different signaling pathways

miRNAs are now considered regulators involved in the HK-II-mediated Warburg effect. A recent study revealed that the miR-155/HK-II axis is an important regulator of tumor plasticity and may be useful for predicting the response and adaptation to aromatase inhibitors in breast cancer patients [122]. According to another study by Jiang et al., miR-155, which is thought to be a pro-inflammatory cytokine, upregulates HK-II through the miR-155/suppressor of cytokine signaling 1 (SOCS1)/STAT3 and miR-155/CCAAT enhancer binding protein β (C/EBPβ)/miR-143 signaling pathways [123].

miR-155/SOCS1/STAT3 signaling pathway

The Warburg effect was strongly increased by mir-155 overexpression and significantly decreased by its knockdown. The HK-II protein level was also dramatically increased by miR-155 and reduced by anti-miR-155. These results suggest that mir-155 increases the Warburg effect by increasing HK-II protein expression.

STAT3 signaling is a major pathway that connects inflammation to cancer. It has been revealed that the long non-coding RNAs (lncRNAs) urothelial cancer-associated 1 (UCA1) promotes glycolysis in bladder cancer cells *via* the mTOR/STAT3/HK-II cascade [124]. In addition, in a recent study, Choe et al. demonstrated an increased correlation between ERp57 and STAT3 in radioresistant laryngeal carcinoma. Furthermore, the inhibition of STAT3 activity using a chemical inhibitor sensitized radioresistant cells to irradiation. These results suggest that the ERp57/STAT3 axis increases the radioresistance of laryngeal carcinoma. This research group also found that an increase in the level of the ERp57-STAT3 complex was associated with a poor prognosis in human laryngeal carcinoma [44].

To explore whether STAT3 directly upregulates HK-II, Jiang et al. used siRNA to knock down STAT3, which caused a significant reduction in the level of HK-II mRNA. Meanwhile, STAT3 inhibition by the addition of JSI-124 (a STAT3 inhibitor) significantly reduced the activity of HK-II. The finding that miR-155 contributes to STAT3 activation *via* suppression of SOCS1 indicates that the miR-155/SOCS1/STAT3 signaling pathway is involved in the Warburg effect [123].

miR-155/(C/EBPβ)/miR-143 signaling pathway

miR-143 directly suppresses HK-II expression in various cancer cells [77, 98, 100, 101]. According to a study by Jiang et al., mir-143 expression was inversely correlated with mir-155 expression in breast cancer cell lines. To explore the mechanisms underlying miR-155-induced activity of miR-143, C/EBPβ (a miR-155 target) was knocked down, which strongly reduced miR-143 expression in ZR-75-30 cells, while the overexpression of C/EBPβ led to an increase in the miR-143 level. In addition, the transfection of miR-155 into ZR-75-30 cells significantly decreased miR-143 expression and C/EBPβ protein levels. This miR-143 inhibition was rescued upon co-expression of a miR-155-resistant form of C/EBPβ [123]. All of these results indicate that the miR-155/(C/EBPβ)/miR-143 signaling pathway is involved in the Warburg effect.

Besides the signaling pathways stated above, there are also other regulators involved in the Warburg effect. SLUG (also known as Snai2) is a zinc-finger transcription factor correlated with poor outcomes in various cancers. In a recent study, Geng et al. demonstrated that curcumin (a major component of the food flavoring turmeric and an anticarcinogenic agent) promoted 4-hydroxytamoxifen (4-OHT) sensitivity of triple-negative breast cancer (lacking ER, PR, and HER2) by inhibiting the SLUG/HK-II signaling pathway [125]. Furthermore, in a study on CA-IX, an enzyme that lowers pH, Yu et al. revealed that the inhibition of hypoxia-induced CA-IX enhanced HK-II inhibitor-induced apoptosis of HCC cells [60].

Although the exact mechanisms underlying HK-II-mediated radioresistance of laryngeal carcinoma have not been fully elucidated, as a number of factors regulating HK-II expression have already been investigated, we speculate that novel inhibitors targeting the HK-II-mediated Warburg effect may emerge as promising tools to enhance the radiosensitivity of laryngeal carcinoma.

## CONCLUSIONS AND THERAPEUTIC PERSPECTIVES

Although RT has been used as a nonsurgical strategy for organ preservation in the appropriate patients with advanced laryngeal carcinoma, resistance to RT remains a major problem, especially for advanced-stage tumors. The Warburg effect has been considered one of the major causes of radioresistance in all kinds of tumor cells, including laryngeal carcinoma. HK-II, which is a key enzyme in the Warburg effect and is positively correlated with the survival of various tumor cells, is highly expressed in laryngeal carcinoma. Numerous agents (e.g., 2-DG, lonidamine, 3-BP, metformin, MJ, oroxylin A, casiopeina II-gly, NA, prosapogenin A, clotrimazole, bifonazole, and anti-HK-II shRNA) have already been revealed to be useful anticancer therapies by targeted inhibiting HK-II or the HK-II-VDAC complex. Thus, we speculate that agents targeted inhibiting HK-II may attenuate the Warburg effect in laryngeal carcinoma and sensitize the cancer cells to RT. The HIF-1α/INPP4B/Akt/mTOR/S6K, PTEN/Akt/mTOR/4E-BP1, miR-155/SOCS1/STAT3, miR-155/(C/EBPβ)/miR-143, and p53/miR-143 signaling pathways, as well as c-Myc and CA-IX, may be involved in the mechanisms elevating HK-II expression in laryngeal carcinoma. Although some studies have indicated that targeted inhibiting the HK-II-VDAC complex may be a promising approach for anticancer therapies, no effective agents have yet reached the clinical stage. Further studies and clinical trials on effective agents targeted inhibiting the signaling pathways of HK-II in laryngeal carcinoma are thus still required. Nonetheless, the findings obtained thus far indicate that targeted inhibiting HK-II may be a novel approach to enhance the radiosensitivity of laryngeal carcinoma.
